# The Deciduous Teeth

**Published:** 1862-03

**Authors:** J. Taft


### THE DECIDUOUS TEETH.

BY J. TAFT.

Read before the Cincinnati Dental Association, Feb. 28, 1862.

In occupying your time for a few moments, I will ask your attention and consideration to the care of the deciduous teeth. This is a subject that has, to a very great extent, been overlooked and neglected, not only by parents, but by physicians, and even by dentists. The opinion is very general, that, as the deciduous teeth are to serve but a temporary purpose—are to be disposed of early in life, they will, without any special care or attention, remain as long as they are required. The idea seems to be common, that attention bestowed upon them is lost, or at least is uncalled for.

Facts in every-day life demonstrate the falsity of this. Very few children retain their temporary teeth in a good condition till they are shed.

We will notice the following three difficulties arising from inattention or neglect of the temporary teeth:

1st. Inefficient mastication.

2d. The pain and suffering resulting from disease of the deciduous teeth.

3d. The influence of this diseased condition upon the permanent teeth.

That mastication with all persons, and under all circumstances, should be perfectly performed, no one will controvert for a moment. It may be supposed that the food upon which a child should subsist, is or should be of such a character as to require but little mastication; and hence that though the teeth are bad and the food imperfectly masticated, yet the child suffers little or no inconvenience from it; this is certainly a false notion, and one in which the gravest consequences are involved. Nature gives no more teeth, and makes them no more perfect than they should be, to accomplish well the work for which they are designed.

Children are oftentimes made to subsist upon food which is not adapted to them, food proper for adults only; which oftentimes requires more vigorous mastication than a child with deciduous teeth can easily perform. For work of this kind, the diseased and imperfect deciduous teeth would avail but little. For the proper preparation of the appropriate food of the child, the teeth should be in good condition and exercised fully. Imperfectly masticated food will prove more deleterious to a child than to an adult; its digestive apparatus has not the same vigor and strength by which to overcome difficulties, nor the same resistance to irritating and injurious agents. Many parents are very much at fault, because they do not train their children to masticate thorougly as soon as they begin to take solid food. This habit, early inculcated, would, in a great many instances, prevent a vast amount of suffering; and doubtless life would be saved in thousands of cases thereby. Cases of indigestion would be very rare indeed in children after they have ceased to receive their maternal subsistence, if their food was of the right kind and well masticated. I would suggest to dentists everywhere, the importance of urging upon parents the necessity of attention to this subject.

The deciduous, as well as the permanent teeth, are far more liable to become diseased, if thrown out of use, or are not employed in legitimate exercise.

We will now briefly consider the pain and suffering which the little patient is subjected to by diseased teeth. In presenting this, we will only embrace the local difficulties, leaving out of view altogether those disturbances more remote, involving pain and suffering—many a time of very grave character—that have their beginning in disease of temporary teeth. The local pain resulting from disease of these teeth is quite diverse in character; the first is from incipient decay, sensitiveness of the dentine, to changes of temperature, and to chemical agents that may come in contact with them, either in food, or otherwise, that will produce decomposition of the tooth structure. Thus by apprehension and actual pain, the process of mastication is interrupted. In more advanced stages of decay, the pulp becomes exposed, irritated and inflamed, and severe and continued pain is the result, oftentimes amounting to an uncontrollable agony.

This frequently occurs early in the life of such teeth, and as a remedy, extraction is the resort. Decay continues, by-and-by the pulp dies, and inflammation of the periosteum and the surrounding tissues supervenes. This is usually accompanied by severe pain, and in many cases is not confined to the immediate vicinity of the tooth, but involves the surrounding parts, to a greater or less extent, according to their susceptibility, extending over the side of the head, the neck and throat in many cases; alveolar abscess is a common result. The suffering and disturbance that accompany these diseased conditions, as we value the comfort and well-being of our children, call for our special attention and best efforts for relief.

We will now for a few moments consider the influence of disease of the temporary teeth upon the permanent organs. The permanent teeth will, doubtless, be better developed if the deciduous perform their proper functions well to the end· The manner in, and extent to which the permanent teeth will be influenced by disease of their predecessors will be governed by the character and extent of that disease.

Some affections will interrupt the process by which the roots of the temporary teeth are removed, and as a result, the growth of the permanent teeth will be checked, or they will be diverted from their proper direction, and so made to occupy a mal-position in the arch.

If the growth of the permanent teeth is interrupted at the proper period for their eruption, there will be a failure in the expansion of the arch; and that at a time, too, when it is most easily accomplished; and hence, in such cases, irregularity occurs, or at least a crowded condition of the teeth results, that is highly inimical to their well-being.

The local irritation and disturbance occasioned by disease of the temporary teeth will, in almost all cases, operate injuriously upon the developing process, going on for the formation of the permanent teeth.

We can not suppose for a moment that in parts immediately surrounded by, and in contact with tissue in a state of inflammation and suppuration, and racked with pain, that so delicate and sensitive process as the elaboration and organization of tooth-bone can be perfectly accomplished. Upon the idea here suggested, you can profitably amplify to a large extent.

Owing to this condition of things, the teeth are far less perfectly formed than they otherwise would have been, and so are far more liable to decay after they have been erupted. The influence of decaying and diseased *temporary* teeth upon the *permanent* ones, already developed, is highly injurious; directly to this point is the language of Mr. Coar. In speaking of the deciduous teeth, he says: "Every practitioner has had frequent occasion to observe the evils consequent upon the early decay of these teeth. Experience has convinced me that the most deleterious effects result from inattention to the teeth of young children; the most serious of which is the direct tendency to promote the disarrangement and destruction of the permanent teeth. This is easily made clear, for it is well known that when caries attacks the temporary teeth, the process of destruction is exceedingly rapid; in fact, it is often found that the crowns of the deciduous molars are entirely obliterated from this cause, even before the eruption of the first permanent molar."

When the carious temporary teeth stand in contact with the permanent, the latter can not be kept clean. Substances lodge upon them, become vitiated, and produce decomposition of the dentine; and also thereby the buccal secretions become vitiated to an extent that proves prejudicial to the teeth; upon this point Mr. Coar remarks: "Now, what are the probable consequences resulting to this new tooth under such circumstances? Experience proves, and it is very obvious, that it is quite impossible to keep this tooth clean so long as the adjoining carious teeth remain in the mouth; and it may be supposed that, like them, it will fall a prey to the same destroying agency. If, then, the deciduous teeth are in a carious condition, the inability to keep the mouth clean affords every facility for the action of such fluids as may be injurious to the teeth; that there are fluids of a decomposing nature in the mouth, the condition of these first teeth affords us abundant evidence; and it can only be supposed that their action will be continued to such organs as are similarly constituted, as those already affected. That this is the case, facts will fully substantiate; for it will be found that when the entire eruption of the first permanent molar has taken place, while the deciduous molars are still remaining, in a carious state in the jaw, this permanent tooth will almost universally become also attacked by caries, and it will often become necessary to extract it, even before the temporary molars have fallen out. This is not the extent of the disastrous consequences, for the early loss of the bicuspids may be traced, in a great measure, to the same cause."

How exactly in accordance with these statements do we find the case to be! In nine-tenths of the cases, the first permanent molars are decayed before the temporary teeth are disposed of; indeed, in a majority of cases, in a very short period after their eruption. Now, in the great majority of cases, this results from the very bad condition in which the temporary teeth have been suffered to remain.

In a few words we may sum up the results of the neglect of children's teeth:

First, Deficient mastication, imperfect digestion, and all the multifarious evils consequent upon it.

Second, Intense and protracted suffering, that will oftentimes involve the whole system to an alarming extent.

Third, Imperfect organization of the permanent teeth, irregularity, early decay, disease and loss.

And now, as a remedy for all this, take *care* of the temporary teeth; keep them in a state of perfect health, from the beginning till they are laid aside by nature, till they have fulfilled their mission. From the first keep them clean, regardless of the effort it may cost. Upon this point we can not do better than quote from the author above cited.

" These teeth should be as carefully examined and as thoroughly treated as those of an adult. Upon the appearance of the first incisors, the child should be accustomed to having them regularly brushed, and if this is diligently attended to, it will in nearly every case prevent the deposit of that 'green tartar,' which is so destructive to these teeth. The child also becomes habituated to the use of the brush, and learns to regard it as one of his daily necessities."

And when decay makes its appearance, if superficial, have it at once removed by the proper skill, but if too deep-seated for this, have it filled, as perfectly as though for an adult. Again we quote: "When the molars are fully developed, they should be examined with care, and if any cavities are found in them, the caries should be entirely removed, and the cavities be entirely filled either with gold or tin foil, an operation which will have the effect of arresting any further decay in these parts. From this time on, the teeth should be examined at intervals of three to six months, and on each occasion treated with as much care as if they were the permanent teeth. Let this course be faithfully pursued, and we shall find that in nearly every case this first set of teeth will be preserved in a sound and healthy condition, until they fall out by the natural absorption of their roots, previous to the eruption of the second teeth. Should this end be accomplished, most important results will follow; the jaws will be expanded to the natural proportions, and much of that irregularity will be prevented, which is now so often met with in the permanent teeth; in addition to this, the delicate enamel, which protects those organs on their first appearance, will not be subjected to the deleterious influences that adjoining carious teeth would be productive of.

By this mode of proceeding, an important moral effect has at the same time been produced on the mind of the young patient; his experience has shown him that the dentist is not a person whose name is only to be associated with all the terrors of "tooth extraction;" and his confidence is established in the beneficial effect to be derived from a proper regard for the advice of his dentist; in after life he will in no case neglect to seek that advice on all necessary occasions.

Parents are undoubtedly much to blame for the indifference with which they regard the children's teeth; for, unfortunately, the dentist is rarely consulted until pain in some of those organs drives the sufferer to him for relief; but I believe that had the profession in general attached the importance to this subject of which it is worthy, our patients would have regarded it as of greater consequence.

